# Highlight selection of radiochemistry and radiopharmacy developments by editorial board

**DOI:** 10.1186/s41181-026-00443-1

**Published:** 2026-03-26

**Authors:** Oliver C. Kiss, Ivan Penuelas, Ana Rey, Zhi Yang, Nic Gillings, Ambi Pillai, Emiliano Cazzola, Raymond M. Reilly, Carlotta Taddei, Jeff Liu, Michael van Dam, Shozo Furumoto, Junbo Zhang, Emerson Soares Bernardes, Filippo Lodi, Winnie Deuther-Conrad, Ya-Yao Huang, Yohannes Jorge Lagebo, Fany P. Ekoume, Miguel A. Avila-Rodriguez, Beverley Summers, Amal Elrefaei, Maria Salgueiro, Catarina F. Ramogida, Peter J. H. Scott

**Affiliations:** 1 Institute of Radiopharmaceutical Cancer Research, Helmholtz-Zentrum Dresden-Rossendorf (HZDR), Dresden, Germany; 2 Helmholtz-Zentrum Dresden-Rossendorf (HZDR), Dresden, Germany; 3 University Clinic of Navarra, Pamplona, Spain; 4 Universidad de la Republica, Montevideo, Uruguay; 5 Peking University Cancer Hospital & Institute, Beijing, China; 6 Copenhagen University Hospital Rigshospitalet, Copenhagen, Denmark; 7 Molecular Group of Companies, Cochin, India; 8 IRCCS Sacro Cuore, Negrar, Italy; 9 University of Toronto, Toronto, Canada; 10 Life Molecular Imaging GmBH, Berlin, Germany; 11 Department of Radiation Medicine, School of Basic Medical Sciences, Peking University, Beijing, China; 12 University of California, Los Angeles, USA; 13 Tohoku University, Sendai, Japan; 14 Beijing Normal University, Beijing, China; 15 Nuclear and Energy Research Institute – IPEN, Sao Paulo, Brazil; 16 IRCCS, Bologna, Italy; 17 Helmholtz-Zentrum Dresden-Rossendorf (HZDR), Leipzig, Germany; 18 Primo Biotechnology, Taipei, Taiwan; 19 University of Addis Abeba, Addis Abeba, Ethiopia; 20 General Hospital & University of Yaoundé, Yaoundé, Cameroon; 21 National Autonomous University of Mexico (UNAM), Mexico City, Mexico; 22 Sefako Makgatho Health Sciences University, Pretoria, South Africa; 23 IAEA and Atomic Energy Authority of Egypt, Cairo, Egypt; 24 University of Buenos Aires, Buenos Aires, Argentina; 25 Simon Fraser University & TRIUMF, Burnaby, Canada; 26 University of Michigan, Ann Arbor, USA

**Keywords:** Highlight articles, Radiochemistry, Radiopharmacy, Radiopharmaceutical sciences, Nuclear medicine

## Abstract

**Background:**

The Editorial Board of EJNMMI Radiopharmacy and Chemistry releases a biannual highlight commentary to update the readership on trends in the field of radiopharmaceutical development and application of radiopharmaceuticals.

**Main body:**

This selection of highlights provides commentary on 25 different topics selected by each co-authoring Editorial Board member addressing a variety of aspects ranging from novel radiochemistry to first-in-human application of novel radiopharmaceuticals.

**Conclusion:**

Trends in radiochemistry and radiopharmacy are highlighted. Hot topics cover the entire scope of EJNMMI Radiopharmacy and Chemistry, demonstrating the progress in the research field in many aspects.

## Background

Each individual co-authoring member of the Editorial Board has selected to highlight an article that has appeared in the radiochemistry, radiopharmacy and imaging agent literature during the period July–December 2025. The aim of this collaborative initiative is to create a biyearly overview for the readers summarizing the latest trends and hot topics in the field.

## Selected highlight articles

### May the clinical trial regulation limit patient’s access to novel therapeutic radiopharmaceuticals?

By Oliver C. Kiss

The preparation of investigational medicinal products (IMPs) to be used in clinical trials has been revised in EU clinical trial regulation 536/2014 (CTR) and came into full force in January 2025, with exemptions for diagnostic radiopharmaceuticals referred to in articles 61 and 63 (Decristoforo et al. 2014). However, therapeutic radiopharmaceuticals are considered as “full” medicinal products. The local impact of the new CTR for radiopharmacies in healthcare establishments in Italy is highlighted in this commentary, as production of (radio)pharmaceuticals in hospitals for use as IMPs in clinical trials has so far been exempted from GMP requirements by national law (Di Iorio et al. 2025). Besides the financial aspects, such as updating infrastructure and personnel to higher standards, the authors also conclude that new opportunities for academic centres by strengthening collaboration between clinicians, researchers, national authorities and industry, may support unmet clinical needs. However, it must be seen as critical that there is now only one academic centre in Italy capable of providing therapeutic radiopharmaceuticals to be used in clinical trials according to CTR standards. This is of particular importance as the current blockbusters in endoradiotherapy, Lutathera® and Pluvicto®, have been developed by academia.

### Unlocking the brain: pretargeted immuno-PET via click chemistry

By Ya-Yao Huang

The blood–brain barrier (BBB) has long constrained the application of antibody-based neuroimaging. Although numerous strategies have been developed to circumvent this limitation, the inherently slow pharmacokinetics of monoclonal antibodies (mAbs) typically require the use of long-lived radionuclides such as zirconium-89 or iodine-124 (De Lucas et al. 2023; Chacko et al. 2013). This dependency introduces several challenges, including suboptimal theranostic matching arising from metal–chelator constraints, compromised image quality due to high-energy γ-emissions, increased radiation exposure from long-lived radionuclides, and added logistical complexity in clinical implementation. These limitations were elegantly addressed by integrating Brain Shuttle technology with ^18^F-labelled bioorthogonal chemistry (Gustavsson et al. 2025). A bispecific antibody targeting both amyloid-β (Aβ) and the transferrin receptor (TfR) was first employed to exploit receptor-mediated transcytosis, thereby enhancing BBB penetration and brain uptake. Subsequently, a two-step pretargeting strategy based on the inverse electron-demand Diels–Alder (IEDDA) reaction was implemented. A trans-cyclooctene-modified “cold” antibody was administered to saturate cerebral targets, followed–after sufficient blood clearance–by injection of a small ^18^F-labelled tetrazine radioligand. This tracer rapidly crossed the BBB and reacted in situ with the antibody within minutes, enabling high-contrast imaging with favourable washout kinetics. This work demonstrates the feasibility of Aβ-specific pretargeted ^18^F-immuno-PET in the brain and highlights click chemistry as a transformative approach for neuroimaging. By decoupling targeting from signal generation, this strategy offers a compelling translational pathway for CNS drug development and therapeutic monitoring, warranting further validation in disease-relevant models.

### Establishing Pb-203 production from electrodeposited Tl targets at Brookhaven national laboratory

By Emiliano Cazzola

Targeted Radionuclide Therapy (TRT) is a rapidly expanding field within nuclear medicine, and a wide range of radionuclides are being investigated for potential clinical application. Among these, lead isotopes are currently under extensive study as promising theranostic agents due to their favourable nuclear properties (Li et al. 2020). Clinical applications of lead-212 have increased significantly in recent years, while lead-203 represents a suitable diagnostic counterpart that could form a matched theranostic pair for SPECT imaging.

As with all clinical translation efforts, isotope availability represents a major bottleneck. While this limitation is largely mitigated for lead-212 through generator-based production, it remains a critical challenge for lead-203 (McNeil et al. 2021). Recently, a comprehensive study of the lead-203 production chain was presented, encompassing thallium target preparation, irradiation studies, target dissolution procedures, and multiple strategies for lead-203 recovery, including impurity characterization and apparent molar activity (AMA) evaluation (Lin et al. 2025). Additionally, an innovative approach to lead-203 formulation using different acetate media is presented, aimed at facilitating radiolabelling with various chelating agents for AMA determination and radiopharmaceutical preparation.

### Get the H outta here! reducing protodemetalation in copper-mediated radiofluorination

By Peter J. H. Scott

**Fig. 1:**
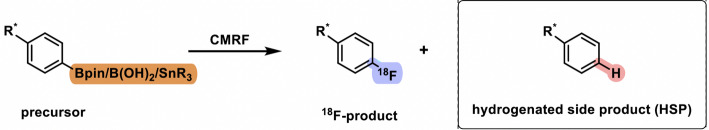
Copper-mediated radiofluorination (CMRF) and common side product resulting from protodemetalation. Reproduced from Kaur et al. under a creative commons attribution 4.0 international license (Kaur et al. 2025)

I might be biased, but I think copper-mediated radiolabelling has opened up chemical space for radiolabelling and fundamentally changed how radiochemists design and synthesize radiopharmaceuticals. Nevertheless, the platform is not without challenges. When we began partnering with the Sanford lab on Cu-mediated radiofluorination (CMRF), and the Gouverneur group was independently developing their version, we all quickly realized that protodemetallation was a competing process (Fig. 1). This not insignificant side reaction gives rise to the corresponding protonated byproduct (Ar**H**) which may, or may not, be readily purified from the desired (hetero)aryl fluoride (Ar^**18**^**F**). Since then, various strategies have been investigated to either separate Ar^**18**^**F** from Ar**H** (e.g. using perfluorophenyl-capped HPLC columns (Mossine et al. 2018) or to mitigate formation of Ar**H** (e.g. ligandless protocol (Sun et al. 2024), but the issue remains incompletely understood.

The recent paper from HZDR investigating factors influencing protodemetalation, as well as the origin of the hydrogen, is thus a welcome addition to the CMRF literature (Kaur et al. 2025). This systematic investigation of factors impacting formation of Ar**H** during CMRF looked at aqueous reaction quenching, use of alcohol co-solvents to improve radiochemical yield (RCY) of Ar^**18**^**F** (e.g. *n*BuOH), type and amount of precursor, base, Cu source and phase transfer catalyst used, as well as reaction time/temperature. Deuterated agents were employed in an effort to identify the source of the proton in Ar**H**. Overall, the authors found that to minimize Ar**H** formation, CMRF conditions should be kept mild (low temperature, short reaction time, low amounts of precursor, base and Cu) and, while use of alcohol co-solvents increase reaction kinetics and improve RCY, they can lead to excessive protodemetalation. Among the precursors tested, -BEpins gave the lowest amount of Ar**H**, while –B(OH)_2_ gave the highest. This work provides valuable insights into the mechanism of CMRF and presents new strategies for minimizing protodemetalation.

### Integrating tumor microenvironment and hypoxia: a bifunctional PET tracer as a paradigm of translational radiopharmacy

By Maria Salgueiro

Recent developments in radiopharmaceutical sciences increasingly demonstrate a shift from single-target imaging toward integrated molecular strategies capable of reflecting tumor heterogeneity. A notable example from the second half of 2025 is the first-in-human evaluation of the bifunctional PET tracer [^68^Ga]Ga-DOTA-NI-FAPI-04, designed to combine fibroblast activation protein (FAP) targeting with hypoxia-sensitive imaging.

**Fig. 2:**
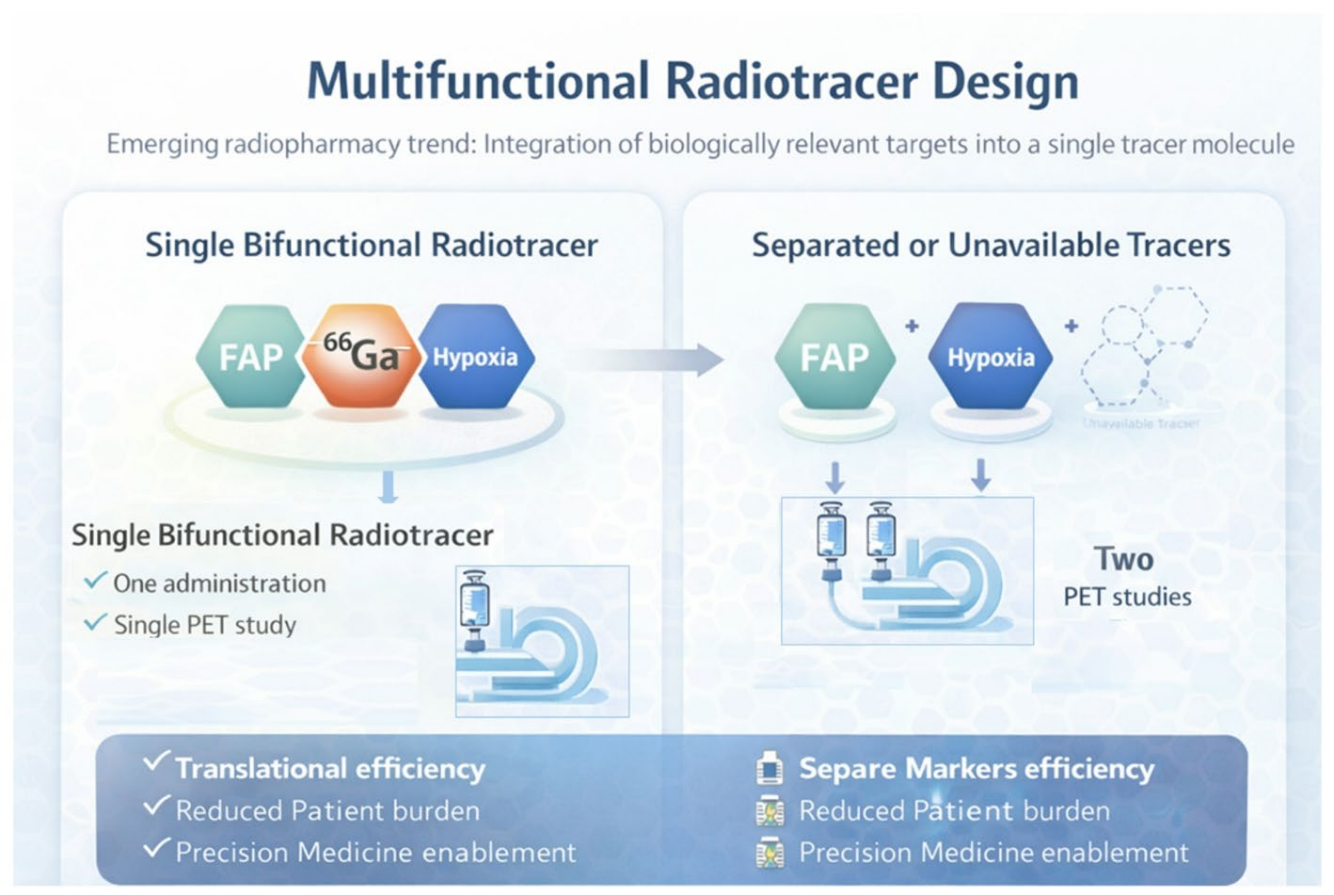
Multifunctional radiotracer strategy integrating complementary biological targets within a single ^68^Ga-labeled imaging agent enables comprehensive biological assessment through one administration and one PET study, compared with separated or partially unavailable tracer approaches that require multiple procedures

Beyond the technical novelty of the tracer, this work (Zang et al. 2025) illustrates a broader evolution in radiotracer engineering, where multiple biologically relevant biomarkers are intentionally integrated within a single molecular construct. By conjugating a FAPI-based targeting vector with a nitroimidazole hypoxia moiety, the authors address two complementary components of the tumor microenvironment—cancer-associated fibroblasts and hypoxic regions—both of which play critical roles in tumor progression, therapeutic resistance, and clinical outcome. Importantly, the first clinical evaluation, including comparison with [^68^Ga]Ga-DOTA-FAPI-04, demonstrates that increased molecular complexity can be translated into human imaging without compromising practical feasibility (Fig. 2).

This study reflects key trends shaping radiopharmacy in the future: the integration of biomarkers, the rational design of bifunctional radiosondes, and a growing emphasis on molecular information relevant for precision medicine. Such approaches not only enhance diagnostic characterization but may also inform patient stratification and treatment planning, particularly in the context of emerging multifunctional theranostic concepts.

Overall, this illustrates how innovation at the level of radiotracer design can directly influence clinical imaging paradigms, reinforcing the role of radiopharmacy as a central driver of personalized nuclear medicine.

### Will astatine-211 become the cornerstone of targeted alpha-therapy?

By Nic Gillings

Astatine-211, a 100% alpha emitter with a half-life of 7.2 h, is receiving increased focus due to its potential for radionuclide therapy applications, also known as targeted alpha therapy. After a slow start, mainly due to limited availability, its potential is being investigated in an increasing number of clinical trials world-wide (Albertsson et al. 2023).

A recent article (Mueller et al. 2026) presents a comprehensive review of astatine-211. Production and isolation methods are described along with a review of current and future production capacities and distribution potential. Advantages, challenges and potential solutions are highlighted with reference to other alpha emitters, such as actinium-225, and the stability of solutions of astatine-211 during storage and distribution. An exhaustive review of ^211^At-astination strategies is included, via nucleophilic and electrophilic routes, along with the utilization of ^211^At-labelled synthons. Lastly, the instability of the carbon-astatine bond is discussed, along with various strategies to reduce in-vivo deastatination, a confounding issue for therapeutic application.

### A step forward to the clinical translation of [^68^Ga]Ga-DOTA-Cys-ATH001 for the non-invasive detection of activated hepatic stellate cells

By Miguel A. Avila-Rodriguez

Liver fibrosis is a chronic disease characterized by a pathological accumulation of extracellular matrix components, potentially leading to cirrhosis or hepatocellular carcinoma. Activated hepatic stellate cells (HSCs) are considered the main source of these accumulations. These cells express platelet-derived growth factor receptor beta (PDGFRß), which is absent in quiescent HSCs, making this receptor a potential molecular target for monitoring liver fibrogenesis.

The molecule ATH001, an affibody developed in 2011, has shown optimal affinity and selectivity for PDGFRβ, and in recent years, several research groups have successfully validated different radiopharmaceuticals based on ATH001 for in vivo PDGFRβ imaging in solid tumors and liver. However, bringing them to clinical applications is not an easy task; the clinical translation of radiopharmaceuticals is a complex, multi-stage journey from bench to bedside, requiring rigorous developments from radiopharmaceutical production, pharmacological characterization, preclinical testing, and clinical trials to get regulatory approval.

A recent publication (Yashaswini et al. 2025) reports on the final steps before clinical translation of [^68^Ga]Ga-DOTA-Cys-ATH001, a potential radiopharmaceutical for the early detection of aHSCs using PET molecular imaging. Single-cell sequencing is a novel approach to further confirm ligand-target selectivity. By doing a great job, this paper is a good example of the rigorous tests a new radiopharmaceutical must undergo to reach the select list of tracers for clinical practice.

### Computationally guided development of a sigma-1 receptor PET tracer for tumor imaging

By Ambi Pillai

This commentary (Kar et al. 2025) highlights a systematic and forward-looking effort to develop a new radiopharmaceutical using computational design as the starting point, an approach that remains relatively uncommon in contemporary radiopharmaceutical research. Instead of relying on traditional trial-and-error synthesis or modification of the existing ligand to obtain a better radiopharmaceutical, advanced molecular docking and simulation techniques were used to identify and optimize a suitable targeting molecule to develop into a radiopharmaceutical. Authors have used this strategy for rapid selection of a promising candidate from commercially available compounds, followed by radiosynthesis and successful biological validation for targeting the sigma-1 receptor (σ1R).

The σ1R is a multifunctional intracellular chaperone protein implicated in cancer progression, neurodegeneration, and cellular stress signalling. Its overexpression in several malignancies, including glioma and melanoma, has positioned σ1R as an attractive molecular target for positron emission tomography (PET) imaging. The development of a novel σ1R-targeting PET radiotracer was started by design using an integrated in silico–to–in vivo strategy (Kar et al. 2025). The work exemplifies how computational screening of commercially available scaffolds can accelerate radiopharmaceutical development while minimizing synthetic burden. Molecular docking and molecular dynamics simulations were employed to identify 1-(4-(4-hydroxyphenyl)piperazin-1-yl)ethanone (PPZ) as a promising σ1R ligand. Importantly, its fluoroethylated analogue, FEt-PPZ, demonstrated improved predicted binding affinity and stable interactions with key residues within the σ1R binding pocket.

Based on the computational insights, the authors developed a simple one-pot, two-step radiosynthetic route for the preparation of [^18^F]FEt-PPZ using an automated synthesis module. The radiotracer was obtained in moderate radiochemical yield with radiochemical purity exceeding 95%, using readily accessible precursors. The streamlined radiosynthesis and compatibility with routine radiopharmacy infrastructure represents notable practical advantages. In vitro evaluation revealed specific uptake of [^18^F]FEt-PPZ in σ1R-expressing U-87 MG (glioma) and B16F10 (melanoma) cells. Competitive inhibition studies using excess non-radioactive ligand confirmed receptor-mediated binding, while minimal cross-reactivity with sigma-2 receptor ligands suggested favourable subtype selectivity. In vivo biodistribution and small-animal PET/CT imaging studies in melanoma-bearing mice demonstrated clear tumour uptake and favourable tumor-to-background contrast. The tracer exhibited predominantly hepatic and renal clearance, consistent with the pharmacokinetic profile of small-molecule σ1R ligands.

The study demonstrates how in silico methods can significantly streamline radiopharmaceutical development, reducing time, cost, and experimental complexity, while improving the likelihood of biological success—an approach with strong implications for the future of targeted molecular imaging as well as therapy.

### Toward in vivo detection of TDP-43 pathology in neurodegenerative disease

By Winnie Deuther-Conrad

Several neurodegenerative diseases, including amyotrophic lateral sclerosis (ALS) and frontotemporal dementia (FTD), and in a subset of cases Alzheimer’s disease (AD), are characterized by mixed pathologies that include the accumulation of TAR DNA-binding protein 43 (TDP-43), a protein involved in RNA processing. Precise characterization of these mixed pathologies is critical for the development of biomarkers, the design of clinical studies, and patient stratification. At present, the presence of TDP-43 pathology can only be definitively confirmed postmortem, limiting the in vivo characterization of mixed pathologies.

A recent paper attempts to address this challenge by identifying two ^18^F-labelled, TDP-43-specific small molecules with high-affinity binding to aggregated TDP-43 (Vokali et al. 2025). They characterized these compounds using multimodal approaches–including high-resolution autoradiography in brain tissue from patients with different TDP-43 proteinopathies, and orthogonal assays such as surface plasmon resonance and radioligand binding–to confirm their specificity and affinity. Furthermore, after confirming their selectivity for TDP-43 over other amyloidogenic proteins such as Aβ and tau, the pharmacokinetics of the compounds were evaluated in a nonhuman primate. Based on the promising results obtained in this study, two potential first-in-class TDP-43 PET tracers, [^18^F]ACI-19,626 and [^18^F]ACI-19,278, are now available for future research. The first-in-human study of [^18^F]ACI-19,626 will be the next critical step to determine whether this agent can be translated into a clinically useful imaging approach.

### An efficient workflow for radiotheranostics production

By Fany P. Ekoume

Research on radiotheranostics is getting more attractive due to reasons including continuous development in the field and extension of the practice to new geographical areas in the world such as the East Africa region.

The current advancements on radiopharmaceuticals for both therapy and diagnostic FAP, as well as promising lead radionuclides, are few examples.

On one hand, the dual role in both diagnostics (with [^68^Ga]Ga-FAPIs achieving high tumor-to-background ratios in various malignancies) and therapy (such as ^177^Lu and ^90^Y-labeled FAP-radiopharmaceuticals showing good result in preclinical and clinical research) makes FAP-binding radiopharmaceuticals promising candidates in cancer treatment (Maes et al. 2025).

On the other hand, lead-212, a radionuclide with a relatively short half-life (10.6 h), offers advantages in terms of dosimetry, including decay in vivo to the alpha emitting bismuth-212 for targeted therapy. More so, lead has a gamma emitting analogue Pb-203 suitable for SPECT imaging leading to an ideal radiotheranostic matched pair very attractive for clinical and preclinical studies (Berckmans et al. 2026).

To sustain the above mentioned and other trends on radiopharmaceuticals, ongoing investigations on the efficiency of radiotheranostics after radiolabelling are also valuable. In fact, a recent study introduced a shortened radio-thin layer chromatography (radioTLC) method to improve the PET radiopharmaceutical production workflow. The aim was to optimize RCP assessment for [^68^Ga]Ga-Edotreotide (Somakit-TOC) by reducing migration distance from 9 to 4 cm. Migration time was analyzed and the evaluation of parameters including specificity, accuracy and robustness showed that alternative method of study offers added benefits. The migration times were significantly reduced with the alternative method (85% reduction). The proposed method enhances PET radiopharmaceutical workflows, allowing faster patient dose preparation without quality loss (Deschavannes et al. 2025).

This approach could be extended to other ^68^Ga-labeled compounds, supporting improved clinical and research applications in nuclear medicine.

### [^99m^Tc]Tc-PSMA-HSG: a next-generation hybrid tracer for precision-guided prostate cancer surgery

By Junbo Zhang

PSMA-targeted radioguided surgery plays a key role in improving the detection of lymph node metastases in prostate cancer, yet currently used tracers rely solely on gamma guidance and lack real-time optical confirmation, limiting surgical precision. Combining fluorescence with radioguided surgery offers a promising solution, although achieving optimal pharmacokinetics and targeting remains challenging.

This study reports the development of a novel dual-modality PSMA-targeted hybrid tracer, [^99m^Tc]Tc-PSMA-HSG, enabling simultaneous radioactive and fluorescence guidance (Schottelius et al. 2025). By incorporating a sulfo-Cy5 fluorophore into the PSMA-I&S scaffold, the tracer preserves high PSMA affinity while integrating complementary imaging signals. Preclinical studies demonstrated superior pharmacokinetic performance compared with [^99m^Tc]Tc-PSMA-I&S, including faster clearance, lower nonspecific background uptake, and approximately 50% higher tumour accumulation in mouse models. In vivo and ex vivo fluorescence imaging in mice using PSMA-HSG demonstrated high tumour-to-background ratios. In a first-in-human study, [^99m^Tc]Tc-PSMA-HSG was well tolerated and enabled accurate intraoperative detection of PSMA-positive lymph node metastases, achieving 100% specificity, sensitivity, and predictive values. Favourable dosimetry further supported its clinical safety. Overall, [^99m^Tc]Tc-PSMA-HSG represents a highly promising hybrid tracer with strong translational potential to advance precision-guided prostate cancer surgery.

### The emerging role of lead-212 in targeted alpha therapy

By Filippo Lodi

Targeted alpha therapy (TAT) with radiopharmaceuticals has emerged as a viable therapeutic option for cancer management. With a short range and high cytotoxicity, α-particles can selectively kill cancerous cells with little impact on surrounding healthy tissue. The therapeutic outcomes of α-emitters in TAT continue to be promising in both preclinical and clinical trials, and they are currently being investigated for a wide variety of malignancies. The achievement of these results is largely attributed to the development of more reliable radionuclide supply chains and improvements in conjugation strategies to enhance safety and productivity.

Among α-emitters, lead-212 has emerged as particularly promising for therapy. Although lead-212 itself is a β^−^-emitter, it acts as an in vivo generator of the α-emitter bismuth-212, combining favourable decay properties with a practical half-life of 10.6 h. The growing role of lead-212 in TAT is described in a review (Scafdi-Muta and Abell 2025) where the recent advances in lead-212 supply and in chelation chemistry are reported. This review highlights novel strategies and approaches that reduce limitations on clinical translation and reports several preclinical and clinical studies targeting receptors such as PSMA, SSTR2, MC1R and HER2 using peptides, small molecules and antibodies. ^212^Pb-based TAT has demonstrated substantial promise in both preclinical models and early clinical studies, and forming a theranostic pair with a γ-emitting ^203^Pb for SPECT imaging makes lead-212 highly attractive for clinical use. Lead-212 has therapeutic potential, but further research is needed to overcome many challenges before it can be used in routine clinical practice.

### Answering long-standing questions about PDMS compatibility with ^18^F-radiochemistry

By Michael van Dam

Microfluidic devices have attracted significant interest in the radiopharmaceutical field due to their extremely compact size, high performance (short synthesis times, high yields, high molar activities), and low reagent consumption. One commonly used microfluidic material – polydimethylsiloxane (PDMS) – has been employed by multiple researchers for production of compounds labelled with fluorine-18, gallium-68, copper-64, and zirconium-89. Over time, there have been some reports on the incompatibility of such devices with fluorine-18 (e.g. device degradation and activity losses); however, such conclusions are not universal, with some reports finding an absence of adverse interactions.

Seeking to understand these contradictory reports, a variety of surface analysis techniques was applied to study the interaction between a PDMS surface and a solution of [^18^F]fluoride and K_2.2.2_/K_2_CO_3_ (commonly used as a phase transfer catalyst) as the liquid is dried by evaporation (McVeigh et al. 2025). Via gas chromatography mass spectrometry studies using headspace sampling, the authors observed the formation of several volatile silicon-containing species, including the fluorine-containing species trimethylfluorosilane. SEM imaging and stylus profilometry studies showed evidence of significant surface etching under the evaporative conditions.

The authors postulated that Si–O bonds in the PDMS are attacked by F^−^ and CO_3_^2−^, a mechanism consistent with both damage to the PDMS, and the loss of radioactivity (via formation of volatile fluorine-containing species). The observation that the degree of damage was correlated with complete drying of the solution could explain how some prior studies could arrive at different conclusions about the suitability of PDMS. Overall, this work seems to have resolved long-standing questions about these microfluidic devices and will guide investigators about the use of PDMS in microsystems for ^18^F-radiochemistry.

### New chelators for Ac-225

By Zhi Yang

Actinium-225 has rapidly emerged as a promising therapeutic radionuclide, driving active research into targeted radiopharmaceuticals. However, conventional chelators such as DOTA present notable limitations–including high labelling temperatures and suboptimal stability of the labelled products–which significantly hinder the development and clinical translation of ^225^Ac-based drugs. In 2025, a series of novel bifunctional chelators (s3p-C-DEPA-NO2, 3p-C-DEPA-NCS, and 3p-C-DEPA-TFP-PEG4) was published (Ketchemen et al. 2025). Among these, s3p-C-DEPA-NO2 demonstrated efficient radiolabelling with a yieldof 93.7 ± 1.4% at 25 °C and reained over 95% radiochemical purity after 6 days of incubation in human serum at 37 °C. Following conjugation to trastuzumab and subsequent ^225^Ac-labelling, the resulting [^225^Ac]Ac-3p-C-DEPA-trastuzumab maintained high radiochemical prity of 91.3 ± 4.3% after 10 days in PBS at 37 °C. Notably, 3p-C-DEPA-TFP-PEG4 proved to be an exceptionally effective bifunctional chelator, achieving excellent radiolabelling efficiency under mild conditions and demonstrating superior in vitro stability relative to DOTA analogues. This study provided a new strategy not only for ^225^Ac-labeling, but also potential BFCs for other radiometal labelling.

### Rate-tuneable, meta-mediated amide bond cleavage for the controlled release of pharmaceuticals

By Caterina F. Ramogida

The highlighted publication describes a tuneable metal-mediated amide bond cleavage (TMAC) strategy that offers significant implications for radiopharmaceutical chemistry (Zhong et al. 2025). The authors demonstrate that incorporating specific amino acid linkers adjacent to a coordinated metal complex enables controlled, metal-triggered hydrolysis of amide bonds under physiological conditions, with kinetics that can be rationally tuned by linker structure and metal coordination environment.

The authors applied this concept to a series of ^68^Ga-labeled PSMA constructs, where TMAC modulates the in vivo release and clearance of radiochelates in a predictable manner. By varying the amino acid linker, the authors achieved differential pharmacokinetics, shifting tracer retention from prolonged circulation and non-target tissue uptake toward more rapid renal clearance while maintaining effective tumour localization. This addresses a long-standing challenge in radiopharmaceutical design: balancing tumour uptake with minimized off-target radiation exposure. Traditional peptide-based radiotracers often exhibit either too rapid elimination or prolonged blood pool activity, leading to suboptimal imaging contrast or increased dose to radiosensitive organs.

Overall, TMAC introduces a chemistry-driven lever to tune radiotracer pharmacokinetics, which could be broadly applied to optimize both diagnostic PET agents and therapeutic radiopharmaceuticals, potentially enhancing safety and efficacy. This work exemplifies how chemical creativity, coupled with a deep understanding of how coordination complexes behave under biological conditions, can drive meaningful innovation in radiopharmaceutical design.

### Extending SV2A PET beyond the brain: [^18^F]SynVesT-1 quantifies synaptic loss in spinal cord injury

By Shozo Furumoto

While the development of novel radiochemical entities remains a cornerstone of our field, the strategic repurposing and methodological extension of radiopharmaceuticals that are clinically characterized and established in neuroimaging are equally important for the continued evolution of nuclear medicine. A timely example of such translational versatility is the recent work by Chen and colleagues (Chen et al. 2025). In this study, the authors extended the use of the synaptic vesicle glycoprotein 2 A (SV2A) radioligand [^18^F]SynVesT-1 beyond its established role in cerebral neuroimaging to enable quantitative assessment of spinal cord pathology.

In vivo PET imaging of the spinal cord is technically demanding, owing to the cord’s small caliber and its susceptibility to motion-related artifacts and partial-volume effects. Using a rat model of spinal cord injury (SCI), these constraints were addressed by implementing a rigorous quantification strategy. By leveraging uninjured cervical segments as an internal reference region, they achieved robust estimation of SV2A-related signal changes. This framework enabled detection of a profound reduction in synaptic integrity (exceeding 50%) at the injury epicenter, consistent with gold-standard immunohistochemistry and complementary protein analyses.

Beyond localized assessment, the authors further used this platform to probe supraspinal consequences of SCI. They reported early SV2A reductions in brain regions implicated in affective and sensorimotor processing, most notably the amygdala and, depending on the reference region, the cerebellum, highlighting that SCI-related synaptic alterations may extend well beyond the spinal lesion itself.

Collectively, this study reinforces a key paradigm: the diagnostic and investigative potential of our existing radiopharmaceutical toolkit is far from exhausted. With rigorous protocol validation and thoughtful expansion to new indications, established tracers such as [^18^F]SynVesT-1 may provide practical, non-invasive biomarkers for SCI, an area in which objective measures of circuit integrity and treatment response remain a major unmet clinical need.

### Convection enhanced delivery of Rhenium (^186^Re) Obisbemeda (^186^RNL) in recurrent glioma: a multicentre, single arm, phase 1 clinical trial

By Raymond M. Reilly

**Fig. 3a:**
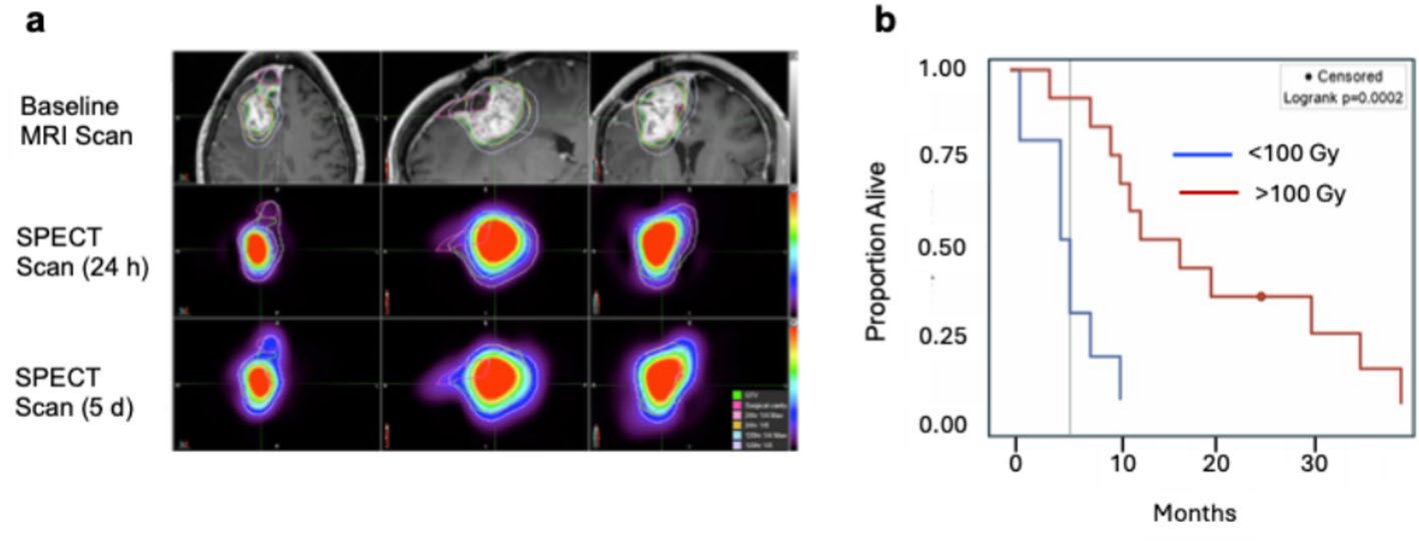
Baseline MRI scans in the axial (left), sagittal (middle) and coronal (right) planes and corresponding SPECT scans at 24 h and 5 d post-CED of ^186^Re-nanoliposomes (^186^Re-obisbemeda). **3b.** Kaplan-Meier survival curves of patients who received < 100 Gy vs. >100 Gy. From: Brenner AJ et al. Nat. Commun. 2025;16(1):279. Adapted and reprinted under creative commons attribution 4.0 international license

Glioblastoma multiforme (GBM) is the most common and lethal brain cancer in adults (Davis et al. 2016). Despite treatment with maximal surgical resection, external radiotherapy and temozolomide chemotherapy (Stupp et al. 2005) almost all patients develop recurrent GBM within 6–9 months (Vaz-Salgado et al. 2023). Recurrent GBM is more difficult to treat, there is no accepted standard-of-care and patients survive < 1 year (Vaz-Salgado et al. 2023). A Phase 1 clinical trial was reported (ClinicalTrials.gov ID: NCT01906385) in 21 patients with recurrent GBM treated by convection-enhanced delivery (CED) of nanoliposomes incorporating [^186^Re]Re-Obisbemeda (Reyobiq^®^, Plus Therapeutics, Houston, TX, USA) (Brenner et al. 2025). Rhenium-186 (t_1/2_=3.7 d) emits β-particles [Eb_max_ = 0.94–1.1 MeV (92%)] that have a 5 mm range and gamma-photons [Eg = 137 keV (9%] imageable by SPECT. CED employs catheters precisely positioned in the brain to infuse therapeutic agents under slight pressure into tumours to achieve convective flow (Kang and Desjardins 2022). SPECT imaging showed focal retention at the site of infusion up to 5 d post-infusion (p.i.) (Fig. 3a) resulting in high radiation absorbed doses up to 740 Gy. Doses > 100 Gy increased median survival to 17 months vs. 6 months in patients who received < 100 Gy (Fig. 3b). There were no serious adverse effects. These results are very promising for treatment of recurrent GBM and build on preclinical studies that showed beneficial effects of CED of these ^186^Re-nanoliposomes in nude rats with U87 human GBM tumours in the brain extending survival to 126 days vs. 49 days for control rats treated with non-radioactive liposomes (Phillips et al. 2012).

### PET of HCC using a radiolabelled antibody fragment targeting glypican-3

By Zhaofei Liu

Hepatocellular carcinoma (HCC) remains a major challenge for clinical PET, largely due to intrinsically high physiological background of the liver, which compromises radiotracer sensitivity and lesion conspicuity. Glypican-3 (GPC3), an oncofetal membrane protein overexpressed in the majority of HCC, has long been recognized as an attractive molecular target. However, reliable and high-contrast clinical PET imaging of GPC3 has proven difficult to achieve, primarily because conventional radiotracers suffer from prolonged hepatic retention and unfavourable pharmacokinetics.

In a recent study, the development of a GPC3-targeted radiotracer was reported based on a ^68^Ga-labeled single-chain variable fragment (scFv), [^68^Ga]Ga-XH-06, and demonstrated its successful translation from preclinical validation to first-in-human PET imaging (Lin et al. 2026). By leveraging the small molecular size and rapid renal clearance of the scFv format, the radiotracer achieves efficient tumour targeting while substantially reducing nonspecific hepatic uptake. Preclinical studies confirmed selective accumulation and robust visualization of GPC3-positive tumours in both subcutaneous and orthotopic HCC models. Importantly, first-in-human PET/MR imaging revealed that [^68^Ga]Ga-XH-06 enables high-contrast detection of HCC lesions, including sub-centimetre tumours, with favourable tumour-to-liver and tumour-to-blood ratios. Beyond improving lesion detection, this work highlights the broader potential of scFv-based radiotracers to overcome intrinsic barriers in liver imaging and positions GPC3 PET as a promising tool for patient stratification and future GPC3-directed theranostic strategies in HCC.

### Turning radiation physics into patient benefit

By Emerson Soares Bernardes

Tumour context and treatment design can widen the therapeutic window of radioligand therapy (RLT) in HER2-low disease (Dewulf et al. 2025). In DLD-1 BRCA2^−/−^ models, adding the PARP inhibitor olaparib increased γH2AX beyond the corresponding single-agent arms for both the β^−^-emitter [^131^I]I-GMIB-2Rs15d and the α-emitter [²²⁵Ac]Ac-DOTA-2Rs15d. With daily olaparib plus an eight-cycle fractionated regimen, both combinations outperformed their respective monotherapies and vehicle control, consistent with synergy.

This matters because RLT discussions often default to a payload ranking, α-emitters outperforming β^−^-emitters, and β^−^-emitters augmented by short-range Auger electrons outperforming β^−^-emitters alone. These data argue that benefit can be dominated by biology and delivery: the ability to repair DNA damage, where the vector localises within tumours, dose-rate, dosimetry, scheduling, and normal-organ limits. Translationally, patient selection should combine functional imaging with biomarkers of impaired DNA repair and, where feasible, longitudinal dosimetry.

The challenge is less conceptual than operational: large head-to-head trials designed primarily to rank radionuclides may be difficult to justify and may divert effort from strategies more likely to move patient-relevant endpoints such as biology-driven combinations, smarter sequencing, and selection frameworks that match treatment to tumour context. The next gains in overall survival will likely not come from radionuclide substitution in isolation, but from integrated biological–radiation systems designed, tested and deployed as combination platforms rather than standalone drugs. As supply diversity increases, the field risks over-indexing on radionuclide novelty and under-investing in the hard work that actually converts radiation physics into patient benefit.

### Quantitative dual-isotope preclinical SPECT/CT imaging and biodistribution of the mercury-197 m/g theranostic pair with [^197 m/g^Hg] HgCl_2_ and a [^197 m/g^Hg]Hg-tetrathiol complex as a platform for radiopharmaceutical development

By Amal Elrefaei

This study delivers an investigation of the commercially available ligand H₄Tetrathiol for [^197m/g^Hg]Hg^2+^ chelation with no carrier added, integrating chelation chemistry, SPECT/CT imaging physics, and quantitative isotope-specific analysis. The authors demonstrate that the acyclic thiol-rich ligand H_4_Tetrathiol efficiently chelates Hg^2+^, achieving exceptionally low ligand-to-metal ratios, including successful radiolabelling at 10^−6^ M, one of the lowest reported for radio-mercury. The resulting [^197m/g^Hg]Hg–Tetrathiol complex exhibits strong in vitro stability against serum proteins, glutathione, and biologically relevant metal ions, while expectedly showing kinetic lability in the presence of excess stable HgCl_2_.

The authors (Randhawa et al. 2025) introduced a comprehensive imaging methodology. Using dedicated phantoms and the XUHS multi-pinhole collimator, the authors achieve sub-millimetre spatial resolution (≥ 1.1 mm) for both 134 keV (^197m^Hg) and 77 keV (^197g^Hg) photon emissions. Their development of HgQuant, a Python-based tool implementing Bateman decay corrections, addresses the inherent complexity of the isomeric transition (IT) relationship between ^197m^Hg → ^197g^Hg and enables reproducible dual-isotope quantification.

The in vivo biodistribution of Hg–Tetrathiol versus HgCl₂ confirm the in vivo stability of the chelated complex, despite its high lipophilicity and liver and spleen uptake and its high lipophilicity. Overall, the paper establishes a solid chemical and quantitative imaging framework for further advancing^197m/g^Hg based theranostics for clinical application.

### Extending the shelf life of ⁶⁸Ge/⁶⁸Ga generators via preconcentration of [⁶⁸Ga]GaCl₃ for preclinical application

By Carlotta Taddei

This study (Mallapura and Eriksson 2025) evaluates and compares with the conventional direct elution method whether using a strong cation exchange (SCX) cartridge to preconcentrate [⁶⁸Ga]GaCl₃ can extend the life of ⁶⁸Ge/⁶⁸Ga generator and improve radiolabelling performance for preclinical applications.

For both methods they used the same 14–18 months‑old generator. With the direct elution the recovery of usable gallium-68 in 500 µL was ~ 30%. A radiochemical yield in the range of 79% was obtained with an apparent molar activity (AMA) of 5.6 MBq/nmol. In comparison with the SCX preconcentration, the entire 5 mL eluate is trapped on a SCX cartridge and eluted in 300–500 µL. The trapping efficiency was > 99% and elution efficiency in the range of 92–95%. The recovery of usable gallium-68 in 500 µL was 95% with a radiochemical yield of 69.0 ± 10.0% and an AMA of 12.6 MBq/nmol, which is doubled the AMA obtained by the direct elution approach.

In summary, preconcentration with SCX cartridges is a robust, cost‑effective method that maximizes gallium-68 recovery and increases tracer AMA, maintaining high radiochemical yield and purity. The preconcentration method adds only 5 min to the total workflow, which is negligible considering the total synthesis time of 28 min. In addition, this method substantially extends the operational life of ^68^Ge/^68^Ga generators delaying costly replacement, which is an especially valuable aspect for preclinical research settings considering the recent advancements of ^68^Ga-tracers (Kleynhans et al. 2024).

### Revolutionary non-invasive RLT in high grade muscle invasive bladder cancer (MIBC)

By Beverley Summers

Bladder cancer is among the most difficult and expensive of cancers to treat – unless the patient is prepared to live without a urinary bladder; not an option chosen by many. Recent advances in immunohistochemistry, linked with AI use of existing datasets, have opened the way to identify patients who can respond to ^177^Lu-radiopharmaceuticals delivered in a new intrathecal approach.

A report on four patients with MIBC G3, is highlighted who could- or would-not undergo neo-adjuvant chemotherapy or cystectomy (Perrone et al. 2025). Based on their molecular profile and immunohistochemical expression of CXCR4 or FAP in tumour samples and PET/CT results, [^177^Lu]Lu-CXCR4 or [^177^Lu]Lu-3BP-3940 was administered in a novel way, via urethral catheter. The radiopharmaceuticals (in doses of 2.9–5.4 GBq) were retained in the bladder for 60–90 min, followed by saline intravesicular flushing and forced diuresis. In two patients, i.v. [^177^Lu]Lu-3BP-3940 was also administered for systemic disease. Safety assessment was on acute and long-term toxicity and imaging response was judged according to the Theranostic Response Criteria in Solid Tumours (THERCIST) scale.

Two patients underwent two intravesicular instillations and achieved remission with no long-term hematological, renal or hepatic toxicity. Two other patients experienced some hematuria but had improved prognosis. The authors concluded that FAP and CXCR4 are promising treatment options for MIBC. The use of the bladder as a radioincubator delivers high radionuclide concentrations directly to the tumour site with minimal systemic effects and excellent tolerability. Patients’ quality of life was favourably affected.

In an *Update on RLT in bladder cancer* presented at the 2026 Theranostics World Congress by Ralph Wirtz, suggested that immunohistochemistry, coupled with the Stratifyer database can be used to predict suitable candidates for this revolutionary intravesicular approach to RLT.

### African radiopharmacy/nuclear medicine on the track of prospective development

By Yohannes Jorge Lagebo

The indispensable components of the nuclear medicine (NM) services are the radiopharmaceuticals (RPs), the imaging facilities and the trained professionals. If any of these is missing, it means running the service is impossible. The advancements in NM are also interlinked with the meaningful developments taking place in these three areas. The African scenario in this regard has been unsteady and remained a source of concerns versus the other Global Regions.

The survey-based investigation conducted by a study group (Mosima et al. 2025) indicated the commencement of some positive changes in enhancing the radiopharmacy/NM services in 24 English-speaking African countries, despite the prevailing challenges. The identified challenges were the unavailability of RPs and NM imaging facilities. 13 countries out of the 24 started NM services with very limited access to RPs and imaging facilities. Besides, the most advanced radiopharmaceutical manufacturing, distributing and utilizing country of the continent (South Africa) belongs to this group. This study highlighted also that some efforts are underway by some of these countries to strengthen the Radiopharmacy/NM services via enhancing access to various radiopharmaceuticals, more advanced imaging facilities and starting the newer NM centres.

Besides, the other more comprehensive and meticulous survey conducted recently, embracing the whole continent by another study group (Brink et al. 2025), reported even more increased signs of the current developmental enhancements in African Radiopharmacy/NM. Those indicative parameters of the developmental progresses have been elaborated in the survey results of the article like RPs, imaging facilities, infrastructure etc. Therefore, the findings from the two studies clearly indicate that the African Radiopharmacy/NM is undergoing a positive move towards meaningful development.

### Synthesis and evaluation of fluorinated peptidomimetics enabling the development of ^18^F-labeled radioligands targeting muscarinic acetylcholine receptor subtype M3

By Ana Rey

Triple negative breast cancer (TNBC) remains a troublesome disease with poor prognosis and limited therapeutic options. In the highlighted paper a new molecular target for TNBC is proposed: the muscarinic receptor M3 and a series of fluorinated radioligands based on the pharmacophore structure of darfenacin, a well know antagonist of muscarinic receptors was designed.

The paper includes the experimental validation of M3 receptor as biomarker in TNBC by transcriptomic analysis of clinical specimens, the structure activity analysis of the fluorinated radioligands and the docking of the best candidate using the crystal structures of the M3 and M2 receptors (Herrera-Rueda et al. 2025). They also performed the radiofluorination using reductive amination chemistry to introduce an appropriate prosthetic group.

The key findings in this paper are: the differential expression of M3 receptors between ER+ breast cancer and TNBC highlighting the promising role of M3 as a novel molecular target in this disease, the incorporation of a glycine-β alanine dipeptide linker to bridge the nonpolar and cationic pharmacophore elements for better receptor interaction, the preparation of a fluorinated radioligand with submicromolar affinity and good selectivity for M3, and the use of computational tools to corroborate affinity and selectivity of the promising candidate.

In conclusion, this study opens a new horizon in the imaging of TNBC that could also aid in future therapy and follow up of this important disease.

### Molecular precision in chemo-radio-theranostics: a chelator-modified, albumin-hitchhiking Evans blue-camptothecin nanoprodrug

By Ivan Penuelas

The highlighted study (Xiong et al. 2025) addresses a longstanding challenge in radiopharmacy: integrating chemotherapy and radionuclide therapy within a single compact molecular entity. A DOTA-modified Evans Blue-camptothecin (EB-CPT) nanoprodrug was synthesized by coupling glutathione-responsive CPT (bearing a disulfide linker) with lysine-functionalized EB bearing a macrocyclic chelator. The amphiphilic conjugate self-assembles into ~ 100 nm nanoparticles, enabling > 99% efficient radiolabelling with therapeutic lutetium-177 or diagnostic gallium-68/copper-64 with excellent radiochemical stability.

**Fig. 4:**
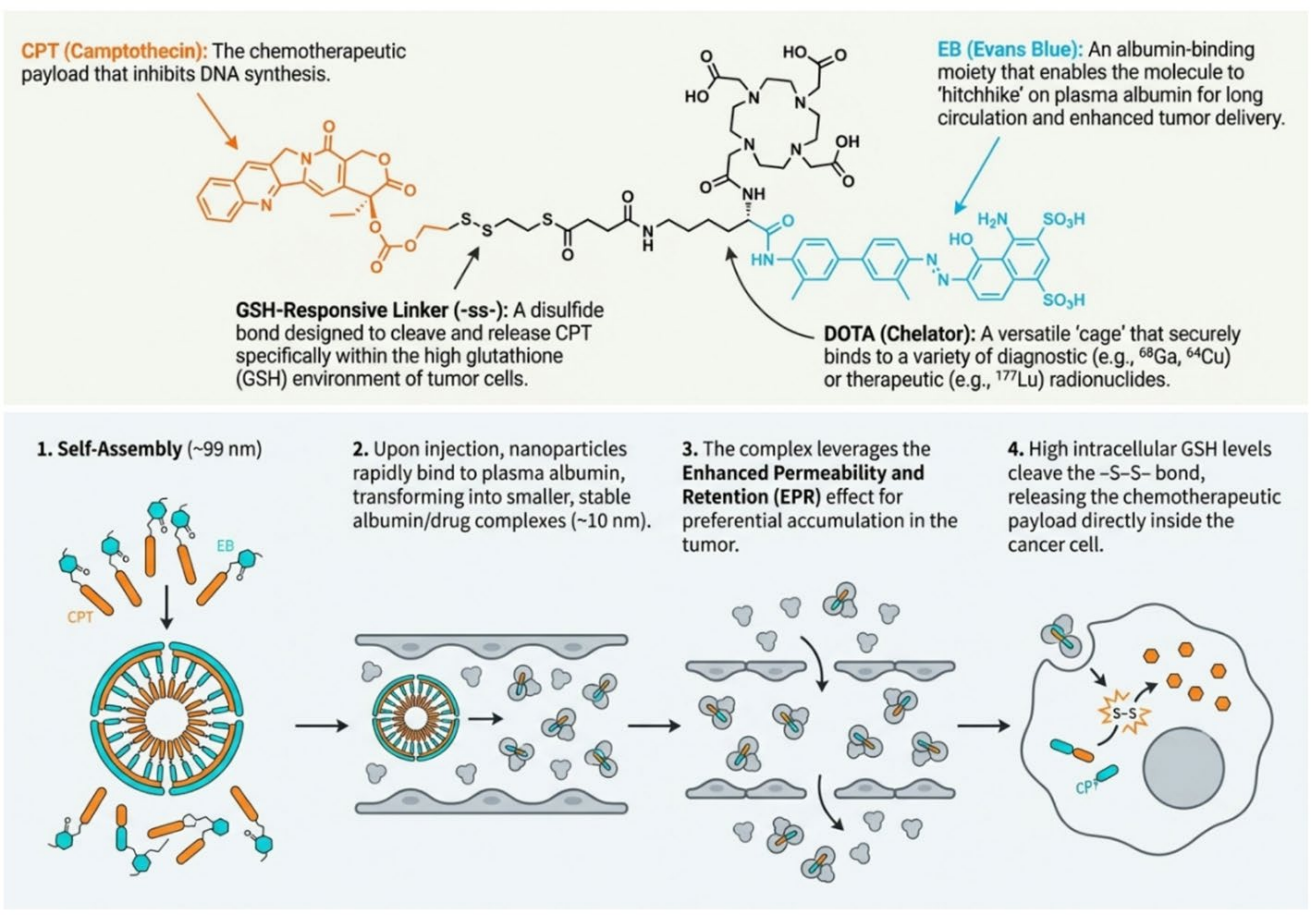
Demonstration of principle of integrating chemotherapy and radionuclide therapy within a single compact molecular entity and self-assembly to form nanoparticles

The chemical innovation resides in a tripartite multimodal design: EB acts as a high-affinity albumin binder, creating long-circulating complexes in vivo; a disulfide linker enables selective, glutathione-responsive CPT (topoisomerase I inhibitor) release in the reducing tumour microenvironment; and the chelator permits flexible radionuclide conjugation without compromising self-assembly. In vitro studies confirm the critical disulfide role, showing markedly higher cytotoxicity versus non-cleavable analogues, validating intracellular prodrug activation. This rational architecture overcomes the pharmacokinetic mismatch inherent to separate chemotherapy and radioligand administration (Fig. 4).

In HCT116 xenografts, single-dose [^177^Lu]Lu-DOTA-EB-CPT (18.5 MBq) plus EB-CPT (5 mg/kg) achieved ~ 88% tumour growth inhibition versus 49% for radionuclide monotherapy and 25% for chemotherapy alone, with peak retention of 5.71%ID/g at 8 h.

First-in-human evaluation of [^68^Ga]Ga-NOTA-EB-ss-CPT in 7 colorectal cancer patients demonstrated excellent safety, tolerability, and favourable dosimetry (2.37 × 10^−2^ mSv/MBq), with progressive tumour enrichment exceeding [^18^F]FDG contrast. This chemically sophisticated construct elegantly balances tumour accumulation, safety, and diagnostic efficacy through rational molecular design, representing a relevant advance toward clinical chemo-radiotheranostics.

## Data Availability

Datasets mentioned in this article can be found in the cited articles.

## Abbreviations

AMA: Apparent molar activity
BBB: Blood brain barrier
BFC: Bifunctional chelator
CMRF: Copper mediated radiofluorination
CTR: Clinical trial regulation
DNA: Deoxyribo nucleic acid
FAPI: Fibroblast activating protein inhibitor
FDA: Food and drug administration
FDG: Fludeoxyglucose
GBM: Glioblastoma multiforme
GMP: Good manufacturing practice
HCC: Hepatocellular carcinoma
HSC: Hepatic stellate cells
IMP: Investigational medicinal product
NM: Nuclear medicine
PDGFR: Platelet derived growth factor receptor
PDMS: Polydimethylsiloxane
PET: Positron emission tomography
RCY: Radiochemical yield
RP: Radiopharmaceutical
RLT: Radioligand therapy
TAT: Targeted alpha therapy
TNBC: Triple negative breast cancer
TRT: Targeted radionuclide therapy

## References

[CR1] Albertsson P, Bäck T, Bergmark K, Hallqvist A, Johansson M, Aneheim M, Lindegren S, Timperanza C, Smerud K, Palm S. Astatine-211 based radionuclide therapy: current clinical trial landscape. Front Med (Lausanne). 2023;9:1076210. 36687417 10.3389/fmed.2022.1076210 PMC9859440

[CR2] Berckmans Y, Kleynhans J, Van Mechelen S, Goffin K, Baete K, Koole M, Coosemans A, Cocolios TE, Deroose CM, Seimbille Y, Cleeren F. Lead radionuclides for theranostic applications in nuclear medicine: from atom to bedside. Theranostics. 2026;16:6. 10.7150/thno.126086 PMC12775822 41510167

[CR3] Brenner AJ, Patel T, Bao A, Phillips WT, Michalek JE, Youssef M, et al. Convection enhanced delivery of Rhenium (^186^Re) Obisbemeda (^186^RNL) in recurrent glioma: a multicenter, single arm, phase 1 clinical trial. Nat Commun. 2025;16:2079. 40055350 10.1038/s41467-025-57263-1 PMC11889265

[CR4] Brink A, Kleynhans J, Grigoryan A, Omar W, Mekonnen BW, Kolade OU, Mokoala K, Sangiwa BA, Hashford F, Nagaraj H, Sakr TM, Bentaleb N, Giammarile F, Estrada-Lobato E, Elrefaei A, Knoll P, Korde A, Paez D. The current status of nuclear medicine in Africa. J Nucl Med. 2025. 10.2967/jnumed.125.271248. 10.2967/jnumed.125.271248 41381238

[CR5] Chacko AM, Li C, Pryma DA, Brem S, Coukos G, Muzykantov V. Targeted delivery of antibody-based therapeutic and imaging agents to CNS tumors: crossing the blood–brain barrier divide. Expert Opin Drug Deliv. 2013;10:907–26. 23751126 10.1517/17425247.2013.808184 PMC4089357

[CR6] Chen B, Zheng C, Balayeva T, Toyonaga T, Wang X, Tong J, Mennie W, Mihailovic J, Coman D, Hyder F, Strittmatter SM, Carson RE, Huang Y, Cai Z. [^18^F]SynVest-1 PET detects SV2A changes in the spinal cord and brain of rats with spinal cord injury. J Nucl Med. 2025. 10.2967/jnumed.124.269291. 40675757 10.2967/jnumed.124.269291 PMC12410297

[CR7] Davis ME, Glioblastoma. Overview of disease and treatment. Clin J Oncol Nurs. 2016;20:S2–8. 27668386 10.1188/16.CJON.S1.2-8 PMC5123811

[CR9] de Lucas ÁG, Lamminmäki U, López-Picón FR. ImmunoPET directed to the brain: a new tool for preclinical and clinical neuroscience. Biomolecules. 2023;13:164. 36671549 10.3390/biom13010164 PMC9855881

[CR8] Decristoforo C, Penuelas I, Elsinga P, Ballinger J, Winhorst AD, Verbruggen A, et al. Radiopharmaceuticals are special, but is this recognized? The possible impact of the new clinical trials regulation on the preparation of radiopharmaceuticals. Eur J Nucl Med Mol Imaging. 2014;41(11):2005–2007. 24990405 10.1007/s00259-014-2838-z

[CR10] Deschavannes A, Terashi K, Piquemal M, Rioufol C, Clotagatide A. Optimization of radiochemical purity assessment for [^68^Ga]^68^Ga-EDOTREOTIDE (Somakit-TOC^®^): a shortened r-TLC method for improved PET radiopharmaceutical workflow. EJNMMI Radiopharm Chem. 2025;10:22. 40358768 10.1186/s41181-025-00341-y PMC12075080

[CR11] Dewulf J, Navarro L, Dumauthioz N, Berdal M, Nagachinta S, Gaspariunaite V, Pombo Antunes AR, Lahoutte T, Massa S, Devoogdt N, D’Huyvetter M. Preclinical synergistic effects when combining a radiolabelled HER2-targeting single-domain antibody with the PARP inhibitor olaparib. J Transl Med. 2025. 10.1186/s12967-025-07572-2. 41390415 10.1186/s12967-025-07572-2 PMC12817464

[CR12] Di Iorio V, Boschi S, Brugunoli E, Sansovini M, Matteuci F, Masini C, Monti M. How regulation 536/2014 is changing academic research with therapeutic radiopharmaceuticals: a local experience. Pharmaceuticals. 2025;18:1709. 41304954 10.3390/ph18111709 PMC12655690

[CR13] Gustavsson T, Kustermann T, Hvass L, Wuensche TE, Clausen AS, Stotz S, Shalgunov V, van den Broek SL, Knudsen GM, Aldana B, Ferri E, Cannazza G, Niewoehner J, Gobbi L, Honer M, Battisti UM, Kjaer A, Herth MM. Pretargeted immuno-PET imaging of amyloid-β in the brain using bioorthogonal click chemistry. Mol Pharm. 2025;22:7600–10. 41198055 10.1021/acs.molpharmaceut.5c01180 PMC12673582

[CR14] Herrera-Rueda MA, Boateng MA, Wuest M, West FG, Wuest F. Synthesis and evaluation of fluorinated peptidomimetics enabling the development of 18F-labeled radioligands targeting muscarinic acetylcholine receptor subtype M3. ChemMedChem. 2025;20:e202500572. 41147419 10.1002/cmdc.202500572 PMC12711157

[CR15] Kang JH, Desjardins A. Convection-enhanced delivery for high-grade glioma. Neurooncol Pract. 2022;9:24–34. 35096401 10.1093/nop/npab065 PMC8789263

[CR16] Kar S, Chakraborty A, Lakshminarayanan N, Rajesh C, Chaudhuri P, Ray MK, Bose K, Banerjee S, Basu S, Mallia MB. Convenient one-pot synthesis of 1-(4-(4-(2-[^18^F]fluoroethoxy)phenyl)piperazin-1-yl)ethanone ([^18^F]FEt-PPZ) for imaging tumors expressing sigma-1 receptors. RSC Adv. 2025;15(29):23943–53. 40642458 10.1039/d5ra02999f PMC12242378

[CR17] Kaur S, Wenzel B, Oehme R, Wiesner C, Kopka K, Moldovan RP. The hydrogenation side reaction in copper-mediated radiofluorination. EJNMMI Radiopharm Chem. 2025;10:60. 40921858 10.1186/s41181-025-00384-1 PMC12417351

[CR18] Ketchemen JP, Ahenkorah S, Nwangele E, Pearl Malaz SS, Ooms M, Cardinaels T, Leekens S, Cleeren F, Fonge H. New chelators for Ac-225. EJNMMI Radiopharm Chem. 2025;10:81. 41428153 10.1186/s41181-025-00408-w PMC12722590

[CR19] Kleynhans J, Ebenhan T, Sathekge MM. Expanding role for Gallium-68 PET imaging in oncology. Semin Nucl Med. 2024;54(6):778–91. 38964934 10.1053/j.semnuclmed.2024.06.001

[CR20] Li M, Sagastume EE, Lee D, McAlister D, De Graffenreid AJ, Olewine KR, et al. 203/212Pb Theranostic radiopharmaceuticals for imaging-guided radionuclide therapy for cancer. Curr Med Chem. 2020;27:7003–31. 32720598 10.2174/0929867327999200727190423 PMC10613023

[CR21] Lin W, Medvedev DG, Cutler CS, Hatcher-Lamarre J. EJNMMI Radiopharm Chem. 2025;10:78. 41369850 10.1186/s41181-025-00403-1 PMC12696209

[CR22] Lin Z, Li M, Pan Z, Hu W, Feng Y, Zhang X, Yin H, Wang S, Song Z, Lv X, Song X, Zheng D, Ruan W, Gai Y, Yang M, Jiang D, Cai X, Zhu J, Lan X et al. Glypican-3-targeted PET imaging for precise diagnosis of hepatocellular carcinoma: from bench to bedside. Clin Cancer Res. 2026;32(3):550-561. 10.1158/1078-0432.CCR-25-1856 10.1158/1078-0432.CCR-25-1856 41020769

[CR23] Maes J, Polet B, Kleynhans J, Van Herpe F, Goffin K, Dekervel J, Nafteux P, Topal B, Cleeren F, Deroose CM. Therapeutic application of fibroblast activation protein (FAP)-binding radiopharmaceuticals: review of opportunities and challenges. Cancers. 2025;17:24. 10.3390/cancers17244019 PMC12731750 41463267

[CR24] Mallapura H, Eriksson O. Extending the shelf life of ⁶⁸Ge/⁶⁸Ga generators via preconcentration of [⁶⁸Ga]GaCl₃ for preclinical application. EJNMMI Radiopharm Chem. 2025;10:76. 41296135 10.1186/s41181-025-00406-y PMC12669415

[CR25] Mc Neil BL, Robertoson AKH, Fu W, Yang H, Hoehr C, Ramogida CF, et al. Production, purification, and radiolabeling of the 203/212Pb theranostic pair. EJNMMI Radioharm Chem. 2021;6(1):6. 10.1186/s41181-021-00121-4 PMC7851237 33527221

[CR26] McVeigh M, Frech C, Lin M, Ta R, Manning HC, Bellan LM. Understanding the Compatibility of Fluoride-Based Radiopharmaceutical Reaction Solutions and PDMS. ACS Appl Mater Interfaces. 2025;18:2775–80. 41424215 10.1021/acsami.5c21729 PMC12781053

[CR27] Mosima LS, Manicum AE, Summers B. Availability of radiopharmaceuticals and imaging equipment in english-speaking African countries. J Nucl Med Technol. 2025;53(Suppl 1):S118–24. 10.2967/jnmt.125.270432 PMC12688044 40925659

[CR28] Mossine AV, Brooks AF, Bernard-Gauthier V, Bailey JJ, Ichiishi N, Schirrmacher R, Sanford MS, Scott PJH. Automated synthesis of PET radiotracers by copper-mediated ^18^F-fluorination of organoborons: importance of the order of addition and competing protodeborylation. J Label Comp Radiopharm. 2018;61:228–36. 10.1002/jlcr.3583 PMC5896751 29143408

[CR29] Mueller M, Pedersen NB, Shalgunov V, Jensen AI, Battisti UM, Herth MM. Astatine-211-towards in vivo stable astatine-211 labeled radiopharmaceuticals and their (pre)clinical applications. Med Res Rev. 2026;46:203–37. 40888104 10.1002/med.70008 PMC12673465

[CR30] Perrone E, Wirtz R, Storz E, Eismant A, Parkar T, Ghai K, Benz-zils D, Hackermüller N, Greifenstein L, Heidenreich A, Baum RP. Revolutionizing bladder cancer treatment: Tolerability and efficacy of intravesical RadioMolecularIncubator Theranostics with ^177^Lu-CXCR4 and ^177^Lu-3BP-3940 in high-grade muscle invasive bladder cancer (MIBC). J NuclMed. 2025;66(1):251926.

[CR31] Phillips WT, Goins B, Bao A, Vargas D, Guttierez JE, Trevino A, Miller JR, Henry J, Zuniga R, Vecil G, Brenner AJ. Rhenium-186 liposomes as convection-enhanced nanoparticle brachytherapy for treatment of glioblastoma. Neuro Oncol. 2012;14:416–25. 22427110 10.1093/neuonc/nos060 PMC3309864

[CR32] Randhawa P, Rodriguez-Rodriguez C, Konir H, Davey PRWJ, Chen S, Radchenko V, Ramogida CF. Quantitative dual-isotope preclinical SPECT/CT imaging and biodistribution of the mercury- 197m/g theranostic pair with [^197m/g^Hg] HgCl_2_ and a [^197m/g^Hg]Hg-tetrathiol complex as a platform for radiopharmaceutical development. EJNMMI Radiopharm Chem. 2025;10:67. 41085959 10.1186/s41181-025-00391-2 PMC12521730

[CR33] Scafdi-Muta JM, Abell AD. ^212^Pb in targeted radionuclide therapy: a review. EJNMMI Radiopharm Chem. 2025;10:34. 40591218 10.1186/s41181-025-00362-7 PMC12214231

[CR34] Schottelius M, Viertl D, Buckle T, Koch H, Martin S, Litvinenko A, Patt M, van Willigen DM, van Leeuwen FWB, Wester HJ, Weckermann D, Liebiech A, Gaeble A, Reitsam NG, Maekle B, Enke JS, Brosch-Lenz J, Lapa C. [^99m^Tc]Tc-PSMA-HSG for PSMA-targeted Hybrid Surgical Guidance: a new addition to the PSMA-I&S/I&T family. EJNMMI. 2025. 10.1007/s00259-025-07623-2 10.1007/s00259-025-07623-2 PMC13013135 41233512

[CR35] Stupp R, Mason WP, van den Bent MJ, Weller M, Fisher B, Taphoorn MJ, et al. Radiotherapy plus concomitant and adjuvant temozolomide for glioblastoma. N Engl J Med. 2005;352:987–96. 15758009 10.1056/NEJMoa043330

[CR36] Sun J, Jaworski C, Schirrmacher R, Hall DG. Suppressing protodeboronation in Cu-mediated ^19^F/^18^F-fluorination of arylboronic acids: a mechanistically guided approach towards optimized PET probe development. Chemistry. 2024;30:e202400906. 38959115 10.1002/chem.202400906

[CR37] Vaz-Salgado MA, Villamayor M, Albarran V, Alia V, Sotoca P, Chamorro J, et al. Recurrent glioblastoma: a review of the treatment options. Cancers (Basel). 2023;15:4279. 37686553 10.3390/cancers15174279 PMC10487236

[CR38] Vokali E, Chevalier E, Dreyfus N, Charmey D, Melly T, Kocher J, Ratnam M, Serra AM, Jaquier T, Delgado C, Ravache M, Scialo C, Cappelli S, Kroth H, Capotosti F, Luthi-Carter R, Afroz T, Derouazi M, Constantinescu CC, Seelaar H, Buratti E, Nelson PT, Polymenidou M, Pfeifer A, Kosco-Vilbois M, Seredenina T. Development of [^18^F]ACI-19626 as a first-in-class brain PET tracer for imaging TDP-43 pathology. Nat Comm. 2025;16:9358. 10.1038/s41467-025-64540-6 PMC12552610 41136425

[CR39] Xiong H, Wang R, Zhang H, Zhang Q, Qin Y, Du C, Zhanf X, Ye J, Shi C, Shen H, Zhu Z, Zhou Z, Chen X, Zhang J. Preclinical and first-in-human study of a compact radionuclide labeled self-assembly nanomedicine for chemo-radio-theranostics of cancer. ACS Nano. 2025;19:3953–65. 39806279 10.1021/acsnano.4c18489

[CR40] Yashaswini C, Mitran B, Papadopoulos N, Wegrzyniak O, Lofblom J, Nordstrom H, Velikyan I, Abouzayed A, Johanson L, Hagmar P, Wagner M, Frejd FY, Korsgren O, Heldin CH, Friedman SL, Erikson O. PDGFRß targeted positron emission tomography as a non-invasive biomarker for activated hepatic stellate cells: lasts steps before clinical translation. EJNMMI Radiopharm Chem. 2025;10:80. 41389114 10.1186/s41181-025-00410-2 PMC12711615

[CR41] Zang J, Cheng H, Luo Y, Jin W, Lai Y, Zheng Q, Peng Y, Kung HF, Zhu L, Ke C, Liu C, Miao W. EJNMMI. First-in-human study of a novel bifunctional PET tracer [^68^Ga]Ga-DOTA-NI-FAPI-04 targeting FAP and hypoxia. 2025. 10.1007/s00259-025-07625-0. 10.1007/s00259-025-07625-0 41168395

[CR42] Zhong Z, Śmiłowicz D, Gork MJ, Garman LC, Guzei IA, Boros E. Rate-tunable, metal-mediated amide bond cleavage for the controlled release of pharmaceuticals. J Am Chem Soc. 2025;10:1021. 10.1021/jacs.5c13166 PMC12636028 41202204

